# Dropsy of the Amnios—Operation, Recovery

**Published:** 1873-01

**Authors:** J. W. B. Reynolds

**Affiliations:** Sacramento


*Dropsy of the Amnios—Operation, Recovery.* By J. W. B. Reynolds, M.D., Sacramento.

On the third of July I was requested to visit Mrs. S., twenty miles distant. She was about thirty-six years of age, of small stature, and the mother of seven children. On inquiry I found she was about five months advanced in pregnancy, but the enlargement of the abdomen was as great as common at the end of seven months. On percussion I found the flat sound of water, but I was at a loss to determine whether the fluid was in an ovarian sac, intraperitoneal, or in the uterus. As she had no appetite, the liver torpid, and kidneys deficient in their secretion, I thought it best to put her on a course of alteratives and diuretics, hoping the improvement in the general health anticipated from this course might arrest the abnormal secretion and keep it in check till the end of her gestation. The general health was much improved by the remedies used, but on the eleventh I was called again to visit her. I now diagnosed dropsy of the amnios. She had enlarged rapidly since my last visit; she was unable to sleep, and was suffering a great deal from pain in the right side and between the shoulders. By means of hot fomentations, painting the abdomen with iodine, and anodynes internally, she passed the time in rather less suffering till the evening of the twenty-first, when I was again sent for. After riding nearly all night to get to the residence of my patient, I found her sitting bolt upright in the bed, in which position she had been for five days and nights—in fact, she could not even lie down to sleep, saying she felt as though she would suffocate whenever she attempted it. She was greatly emaciated, and the abdomen was so full and tense that the respiration seemed almost suspended, and there seemed little hope of the poor woman being able to stand it many more hours without some relief. I had informed the husband several days previously what would probably have to be done, and as she had been made aware of it, she was urgent for the operation.

Accordingly I proceeded at once to attempt the dilatation of the os uteri by the gradual, but pretty forcible, insertion of the index finger. Then applying the extract of belladonna pretty freely within the cervical canal—applying a tampon and administering the fluid extract of ergot twice—about two-thirds of a drachm at a time, one hour apart, I had the pleasure of finding, upon examination, that the os was dilated as large as a quarter of a dollar. By encouraging her now with the hope of almost immediate relief, I prevailed on her to lie down until I could introduce the instrument to cut the membranes. I used a blunt-pointed pair of scissors, curved on the flat, guiding them through the vagina and into the uterus on my finger. They answered my purpose admirably, and I found no difficulty in cutting the membrane which contained the fluid. There was upwards of thirty-two pints of fluid, resembling the liquor amnii, discharged. My patient was asleep before it was half discharged; and after sleeping comfortably for an hour, labor pains commenced, and in about one hour more she gave birth to the child, which was nearly six months grown. It gave a slight cry, a gasp or two, and expired. The secundines were easily removed. But in removing the placenta, I felt a tumor nearly as large as the fist attached to it. When I removed it where I could examine it, I found the tumor filled with semi-coagulated blood, about four ounces in quantity. It was enclosed within a membranous sac which appeared to be formed by a portion of that part of the amnios which is reflected over the internal face of the placenta. Whether this unnatural formation had anything to do with the abnormal secretion, I am unable to say. Indeed, it may have been of recent formation.

Cazeaux, in his *Midwifery and Diseases of Pregnancy*, mentions a case from Duclos, of which this was an almost exact counterpart; the rapid emaciation, the great distension of the womb, producing such enormous enlargement of the abdomen, that there was absolutely no room for the play of the diaphragm. She could not even bear any support, saying that the least compression on the back or chest stopped her breathing. So the poor woman sat for five days and nights without support, and almost without sleep or food. As Cazeaux says, diuretics had no effect on the progress of the disease. I had no suitable means to puncture the membranes, and I thought it best to have the os well dilated before operating. Cazeaux says, "If the neck were obliterated, it might become necessary to make a puncture by the vagina near the uterine orifice," and that Camper and Scarpa even advise puncturing between the umbilicus and pubes. I must confess that I cannot conceive of conditions which would render such a proceeding necessary. If he had said, "if the os were entirely obliterated," I could understand the necessity of making a new opening; but where the neck is obliterated, as in the last months of pregnancy, I think the os would only be the more easily dilated. And my success in this case gives me confidence to believe that I could succeed in almost any other without puncturing, and unless the os were completely closed by firm adhesion. This lady had passed an unusual quantity of liquor amnii at her last confinement two years previously, but the quantity had never been so great as to cause any inconvenience during gestation. She recovered her health rapidly after the operation.—*Pacific M. & S. Jour.*

